# Can accelerometry identify/monitor neuropsychiatric symptoms correlated with motor activity in persons living with dementia? Results from a diagnostic test accuracy systematic review

**DOI:** 10.1002/alz.090888

**Published:** 2025-01-09

**Authors:** Elena Guseva, Andrea Iaboni, Krista L. Lanctôt, Nathan Herrmann, Zahinoor Ismail, Amer M. Burhan, Genevieve Gore, Dallas Seitz, Sanjeev Kumar, Marie‐Andrée Bruneau, Andrew Lim, Machelle Wilchesky

**Affiliations:** ^1^ McGill University, Montreal, QC Canada; ^2^ University of Toronto, Toronto, ON Canada; ^3^ KITE Research Institute, Toronto Rehabilitation Institute ‐ University Health Network, Toronto, ON Canada; ^4^ Sunnybrook Health Sciences Centre, Toronto, ON Canada; ^5^ Temerty Faculty of Medicine, University of Toronto, Toronto, ON Canada; ^6^ Neuropsychopharmacology Research Group, Sunnybrook Research Institute, Toronto, ON Canada; ^7^ Hotchkiss Brain Institute, University of Calgary, Calgary, AB Canada; ^8^ Ontario Shores Centre for Mental Health Sciences, Whitby, ON Canada; ^9^ University of Calgary, Calgary, AB Canada; ^10^ University of Montreal, Montreal, QC Canada; ^11^ Centre for Research in Aging ‐ Donald Berman Maimonides Geriatric Centre, Montreal, QC Canada; ^12^ McGill University, Department of Family Medicine, Montreal, QC Canada; ^13^ Lady Davis Institute for Medical Research, Montreal, QC Canada

## Abstract

**Background:**

Wearable sensor technology shows promise for neuropsychiatric symptoms (NPS) identification/monitoring in persons living with dementia (PLWD). Accelerometry measures acceleration of body segments and physical activity. This study explores the diagnostic test accuracy (DTA) of accelerometry to identify the following 6 NPS associated with motor activity: aberrant motor behavior (AMB), aggression, agitation, anxiety, apathy, and wandering.

**Methods:**

As part of a larger systematic review, we conducted an extensive literature search in nine health science and engineering databases. Meta‐analysis involved converting correlations to Fisher's Z scores, calculating 95% confidence intervals using the R Studio Metacor package, and assessing fixed effects, DerSimonian and Laird's random effects models, and heterogeneity.

**Results:**

Out of 12,853 identified records, 84 reports were retained for analysis. Eight reports that provided 12 datasets assessed the DTA of accelerometry data for identifying/monitoring apathy (n=4), AMB (n=1), anxiety (n=2) agitation (n=5), aggression (n=2), and wandering (n=2). Studies included one single‐blinded trial, one non‐randomized trial, and ten observational studies. A random‐effect meta‐analysis yielded a pooled correlation across studies of r=0.61 (0.50; 0.70), heterogeneity I^2^=68%. When the wandering NPS subgroup was eliminated from analysis, heterogeneity fell considerably to I^2^=36%, with a pooled correlation of r=0.55 (0.47; 0.62).

**Conclusions:**

These findings suggest that accelerometry consistently provides good to moderate DTA for identifying/ monitoring NPS associated with motor behavior in PLWD. Future research should prioritize standardizing the reporting of measurement procedures, signal thresholds, outcome measures, devices, and reference standards.

